# Synergistic Effects of Bioactive Compounds on Human Adiposity Mechanisms of Fat Loss and Fat Accumulation

**DOI:** 10.1002/fsn3.71061

**Published:** 2025-10-07

**Authors:** Mohamed Ibrahim Younis, Rawaa H. Tlay, Ammar B. Altemimi, Zheng Ruan, Tarek Gamal Abedelmaksoud

**Affiliations:** ^1^ Food Science Department Faculty of Agriculture, Cairo University Giza Egypt; ^2^ Department of Food Science College of Agriculture, Damascus University Damascus Syria; ^3^ Department of Food Science College of Agriculture, University of Basrah Basrah Iraq; ^4^ State Key Laboratory of Food Science and Resources Institute of Nutrition, Nanchang University Nanchang China

**Keywords:** adiposity regulation, bioactive compounds, cachexia and sarcopenia, fat metabolism, obesity management, synergistic effects

## Abstract

Adipose tissue plays a crucial role in regulating metabolic health in humans, where both an excess and a deficiency can lead to chronic diseases. Bioactive compounds derived from food like polyphenols, alkaloids, terpenoids, peptides, and fibers have been recognized as significant influencers of fat metabolism. They operate through various molecular and systemic pathways, facilitating either adiposity reduction or fat accumulation based on the physiological context. This review consolidates recent progress in comprehending the mechanisms through which these compounds affect lipolysis, adipogenesis, thermogenesis, and appetite regulation. We emphasize the collaborative effects that enhance bioactive efficacy and examine findings from clinical trials focused on obesity, cachexia, and sarcopenia. For example, co‐administration of curcumin with piperine increased curcumin bioavailability up to 20‐fold, and epigallocatechin gallate (EGCG) combined with caffeine modestly enhanced 24‐h energy expenditure by 4% in humans. Similarly, omega‐3 fatty acids combined with vitamin D supplementation improved lean mass by 1.2 kg in sarcopenic adults in recent meta‐analyses. New platforms in personalized nutrition—combining genomics, microbiome analysis, and AI‐driven meal planning—present exciting opportunities for tailored applications. In conclusion, we address significant translational challenges and prospective pathways, focusing on enhancing bioavailability, standardizing outcomes, and ethically expanding personalized interventions. This work highlights the promise of bioactives as effective means for influencing adipose biology and enhancing metabolic health.

## Graphical abstract

Chronic diseases are associated with imbalances in adipose tissue, which plays a crucial role in regulating metabolic health. Food‐based bioactive substances (such as polyphenols, terpenoids, peptides, and fibers) impact fat metabolism by influencing the processes of lipolysis, adipogenesis, thermogenesis, and appetite control. Despite issues with bioavailability and standardization, recent developments demonstrate their synergistic effects, clinical potential in obesity and muscle‐wasting disorders, and opportunities in personalized nutrition.

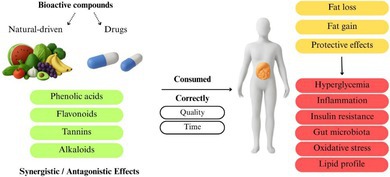

## 1 Introduction

Obesity and metabolic syndrome have emerged as significant global health challenges, largely fueled by a persistent positive energy balance and a lack of physical activity. In recent decades, the incidence of overweight individuals has approximately doubled, with around one‐third of the global population currently categorized as overweight or obese (Ataey et al. 2020; Parfenteva et al. 2024). Excess body weight elevates the likelihood of developing type 2 diabetes, cardiovascular diseases, various cancers, and other long‐term health issues, thereby representing a significant challenge in terms of health complications and mortality rates (Piché et al. 2020). Conversely, pathological wasting conditions, including malnutrition, sarcopenia, and cachexia, manifest in chronic diseases like cancer, heart failure, or infections, resulting in significant loss of adipose and muscle tissue (Berriel Diaz et al. 2024). Cancer cachexia represents a multifaceted systemic syndrome characterized by unintentional weight loss and catabolic processes, which notably deteriorate patient outcomes. In a similar vein, malnutrition and sarcopenia are frequently observed in older adults or those with chronic illnesses (Bullock et al. 2024). Consequently, the “dual burden” of adiposity reduction and fat accumulation illustrates the complex interplay of energy homeostasis dysregulation, which carries significant health implications. Dietary bioactive compounds present a potential avenue for influencing these contrasting metabolic conditions. Foods and extracts encompass a diverse range of phytochemicals and nutraceuticals, including polyphenols like flavonoids, stilbenes, and phenolic acids; alkaloids such as xanthines, including caffeine and capsaicinoids; terpenoids like ginsenosides; non‐digestible fibers; and bioactive peptides, all of which influence energy balance. A number of these compounds have demonstrated effects on adipose tissue function and systemic metabolism in preclinical models. For example, compounds like resveratrol, quercetin, and catechins have been shown to impede the differentiation of adipocytes while also decreasing inflammation and insulin resistance (He et al. 2024). Dietary alkaloids and related compounds, such as caffeine and capsaicin, promote thermogenesis and lipolysis. Meanwhile, soluble fibers influence gut hormones and microbiota, contributing to increased satiety and improved insulin sensitivity (Fernández‐Cardero et al. 2024; Lou et al. 2024). In a similar vein, small peptides obtained from food sources, such as those from milk or soy, can function as signaling molecules, influencing appetite suppression and modifying lipid absorption and metabolism (Hajfathalian et al. 2025). In conclusion, fruits, vegetables, herbs, and various other foods offer a diverse array of polyphenols, alkaloids, terpenoids, fibers, and peptides that collectively have the potential to transform adipose tissue into a leaner, more metabolically active condition (Shin et al. 2024). The ways in which these bioactives influence adiposity reduction or fat accumulation are varied. A variety of compounds facilitate the process of lipolysis, which is the breakdown of fat, while simultaneously hindering adipogenesis, the formation of fat cells, in white adipose tissue (He et al. 2024). Various factors promote the browning of white adipocytes and stimulate brown fat thermogenesis through mechanisms like AMP‐activated protein kinase/silent mating type information regulation 2 homolog 1 (AMPK/SIRT1) and peroxisome proliferator‐activated receptor gamma coactivator 1‐alpha (PPARγ‐PGC1α), resulting in heightened expression of uncoupling protein‐1 (UCP1) and enhanced energy expenditure (Hui et al. 2020). For instance, resveratrol has demonstrated the ability to activate a gut microbiota–bile acid–TGR5‐UCP1 axis, which enhances brown adipose tissue activity and encourages the development of beige adipocytes (Shin et al. 2024). On the other hand, bioactives have the potential to affect appetite and nutrient absorption: they might promote the release of incretins (glucagon‐like peptide‐1 [GLP‐1], peptide YY [PYY]) and gut hormones from enteroendocrine cells or modify sensory signals in the brain, consequently leading to a decrease in food intake (Suryaningtyas et al. 2025). Dietary fibers undergo fermentation by gut microbes, resulting in the production of short‐chain fatty acids that enhance intestinal barrier function and activate satiety signaling pathways, such as those mediated by G‐protein‐coupled receptors (GPR41/43) (Deehan et al. 2022). The gut microbiota plays a crucial role as a mediator: numerous polyphenols and other phytochemicals alter microbial communities (for instance, enhancing Akkermansia or butyrate‐producing bacteria) in ways that promote leanness and enhance insulin sensitivity (Anhê et al. 2015; Chen et al. 2016). In conclusion, bioactive compounds from food interact with various metabolic pathways—including the adaptability of adipose tissue, thermogenic processes, lipase function, hormonal regulation of appetite, and the ecology of gut microbes—to affect the equilibrium between fat storage and fat oxidation. It is important to highlight that these varied compounds frequently engage in synergistic interactions. Integrating bioactives can enhance advantages that surpass their singular impacts. For instance, the intake of coffee offers both chlorogenic acids and caffeine, which collectively enhance lipid metabolism and glucose homeostasis more effectively than either component alone (Nguyen et al. 2024). In a similar vein, the concurrent use of a lipase inhibitor (orlistat) alongside polyphenol‐rich extracts results in a combined effect that enhances the reduction of fat absorption, enabling the use of lower doses and minimizing side effects (Uuh Narvaez et al. 2025). Overall, recognizing multi‐compound combinations can lead to more effective interventions for obesity (or cachexia) at acceptable doses (Chaachouay 2025). This principle of synergy forms the foundation of both traditional dietary patterns, such as the Mediterranean diet, and contemporary nutraceutical approaches. The mechanistic insights presented here hold significant translational relevance. Studies involving human subjects on diets rich in polyphenols, fiber supplementation, and bioactive mixtures have indicated slight enhancements in body composition and metabolic indicators (Zhang, Balasooriya, et al. 2023). For example, studies involving polyphenol‐rich coffee blends or green tea extracts in overweight adults demonstrate decreases in body fat percentage, fasting glucose, and triglycerides (Nguyen et al. 2024; Saud and Salamatullah 2021). Nonetheless, the clinical effects appear to be minimal, underscoring the difficulties associated with bioavailability and the variability observed in human responses. Nevertheless, the idea of utilizing dietary bioactives—particularly in combination—presents a promising complement to lifestyle and pharmacological strategies. In conclusion, a comprehensive understanding of polyphenols, alkaloids, terpenoids, fiber, and peptides, along with their synergistic interactions, could inform innovative nutritional or drug‐nutrient approaches to combat both obesity and cachexia in humans.

## 2 Objective and Methods

The purpose of this review was to summarize updated knowledge of bioactive compound synergy in the modulation of fat metabolism with specific focus on weight management. We performed a systematic literature search in the PubMed, Scopus, Web of Science, and Google Scholar databases using various combinations of bioactive compounds related search terms alongside adiposity or fat metabolism or obesity intervention together with reference screening. The criteria were based only on studies about food or natural bioactives that provided a mechanism of action, evaluated synergy, and were peer‐reviewed; food bioactives not involving explicit molecular targets and lacking mechanistic evidences, as well as non‐peer‐reviewed articles, were excluded. Studies were from various geographical areas and included animal models and human studies. For data analysis, molecular mechanisms (AMPK/SIRT1 activation, PPARγ modulation, thermogenesis, gut–brain signaling), clinical outcomes (body fat percentage, lipid profile, lean body mass), and synergic combinations (phytochemical–phytochemical; phytochemical‐alkaloid; prebiotic–probiotic) were also considered. In all, approximately 240 studies were selected and classified into thematic sections on reduction of adiposity, mechanisms governing fat accumulation, and interactive effects, future perspectives.

## 3 Food‐Derived Bioactive Compounds: Mechanisms and Sources

**FIGURE 1:**
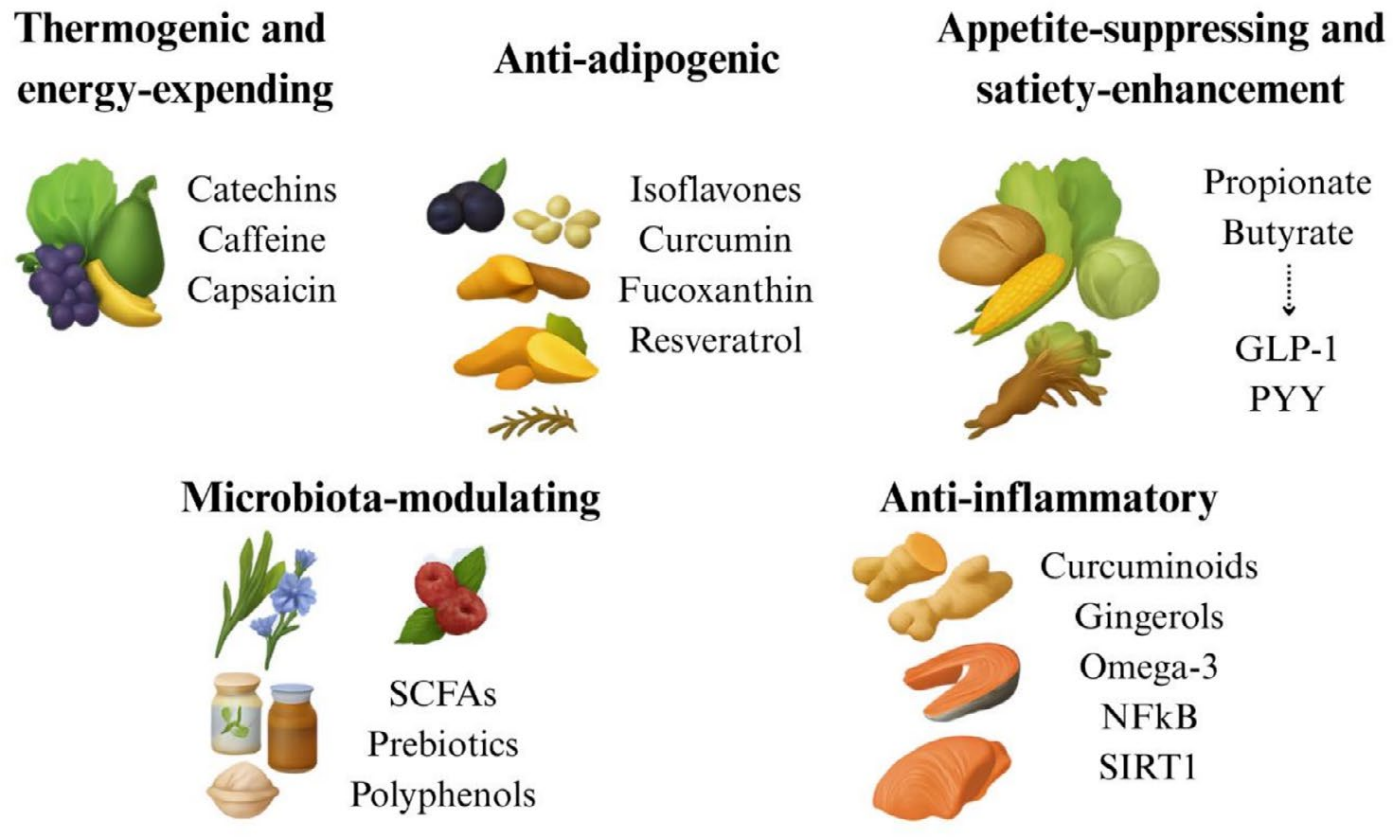
Bioactive sources and mechanisms supporting fat modulation.

Bioactive molecules derived from nature have the potential to impact fat metabolism via various mechanisms (Figure 1). These are categorized based on their primary functions—thermogenic/energy‐expanding, anti‐adipogenic, appetite‐suppressing, lipolytic, microbiota‐modulating, or anti‐inflammatory—along with important food sources. In each category, we emphasize key compounds and their respective cellular targets. The discussion is limited to substances derived solely from diet, including plants, marine foods, fermented products, or microbial metabolites of fiber.

### 3.1 Thermogenic and Energy‐Expending Compounds

Some potent and stimulating phytochemicals increase the basal metabolic rate by triggering thermogenesis in brown/beige adipose tissue or promoting mitochondrial uncoupling. For instance, capsaicin, derived from chili peppers, acts as a transient receptor potential vanilloid 1 (TRPV1) agonist that directly enhances uncoupling protein 1 (UCP1) expression and promotes mitochondrial biogenesis in adipocytes (Takeda and Dai 2022). Similarly, green tea catechins, particularly epigallocatechin gallate (EGCG), along with caffeine derived from coffee and tea beans, stimulate AMP‐activated protein kinase (AMPK) and various pathways that enhance brown adipose tissue activity and promote the browning of white fat (Wang et al. 2024). These compounds enhance lipid oxidation and increase heat production; long‐term consumption is associated with elevated energy expenditure and fat loss in both animals and humans. For example, EGCG not only suppresses lipogenesis but also promotes sympathetic thermogenesis and improves insulin sensitivity (Choi et al. 2020; Oruganti et al. 2023; Zhou et al. 2018). Additional plant thermogenics consist of sulforaphane and mustard oil (allyl isothiocyanate) derived from Brassica vegetables, which activate cold‐receptor transient receptor potential ankyrin 1 (TRPA1) pathways, as well as gingerols found in ginger root that could slightly enhance fat oxidation. In conclusion, compounds such as capsaicins, catechins, and caffeine derived from herbs, tea, and coffee interact with UCP1/AMPK/TRP channels to enhance energy expenditure and promote the utilization of fat stores.

### 3.2 Anti‐Adipogenic and Adipocyte‐Differentiation Inhibitors

A significant group of plant polyphenols and associated compounds inhibit the development of new fat cells or decrease lipid accumulation in adipocytes. Notable instances comprise resveratrol found in grapes and berries, isoflavones like genistein present in soybeans and other legumes, curcumin derived from turmeric root, fucoxanthin sourced from brown seaweeds, gingerols from ginger, and EGCG from green tea. Mechanistically, numerous compounds reduce the activity of key adipogenic transcription factors (peroxisome proliferator‐activated receptor gamma [PPARγ], CCAAT/enhancer‐binding protein alpha [C/EBPα]) and enzymes involved in lipogenesis (fatty acid synthase [FASN], sterol regulatory element‐binding protein 1c [SREBP‐1c]). For instance, fucoxanthin has demonstrated the ability to inhibit PPARγ/C/EBPα in preadipocytes and to induce UCP1 in white fat, thereby promoting beige fat (Ding et al. 2025). In a similar manner, 6‐gingerol inhibited the differentiation of adipocytes by reducing the expression of SREBP‐1 and PPARγ in fat cells (Hong, Um, et al. 2023). The administration of resveratrol in obese mice led to a decrease in weight gain and visceral fat, accompanied by the downregulation of PPARγ2, C/EBPα, SREBP‐1c, and other associated adipogenic genes within adipose tissue (Kim et al. 2011). A significant number of these compounds enhance insulin sensitivity and decrease ectopic lipid storage. In conclusion, fruits, vegetables, and legumes that are high in polyphenols (such as berries, grapes, soy, turmeric, and algae) produce compounds that inhibit adipogenesis and triglyceride synthesis, thus reducing fat accumulation.

### 3.3 Appetite‐Suppressing and Satiety‐Enhancing Compounds

Specific dietary elements enhance feelings of fullness or influence the appetite signals between the gut and brain. Well‐documented instances include fermentable fibers and resistant starches sourced from legumes, whole grains, and vegetables, which are transformed by gut microbes into short‐chain fatty acids (SCFAs). Short‐chain fatty acids like propionate and butyrate interact with enteroendocrine receptors (FFAR2/3) located on intestinal L‐cells, leading to the secretion of GLP‐1 and PYY hormones that help decrease food consumption (Mansuy‐Aubert and Ravussin 2023). Recent studies indicate that propionate significantly elevates circulating PYY/GLP‐1, which in turn slows gastric emptying and enhances feelings of satiety (Caengprasath et al. 2020). Furthermore, short‐chain fatty acids have the capacity to exert effects centrally through the vagus nerve or to traverse the blood–brain barrier, as seen with acetate in the hypothalamus, thereby reducing appetite (Cook and Mansuy‐Aubert 2022). Additional foods that are believed to have appetite‐suppressing effects consist of high‐protein legumes, which can slow digestion and elevate GLP‐1 levels, as well as certain spice compounds. For example, capsaicin and specific pepper alkaloids may modestly diminish hunger signals by temporarily activating nerves. In practical applications, the majority of appetite‐modulating bioactives are facilitated through the fermentation of fiber. Consequently, fruits, vegetables, whole grains, and fermented foods play a role in indirectly reducing intake by nourishing SCFA‐producing microbes—a mechanism derived from the microbiota that contributes to the reduction of fat accumulation.

### 3.4 Microbiota‐Modulating Metabolites

In addition to SCFAs, various molecules derived from diet play a crucial role in shaping gut flora and its metabolic outputs, thereby influencing fat balance. Prebiotic oligo‐ and polysaccharides such as inulin, pectin, and resistant starch found in legumes, onions, chicory, and whole grains enhance the populations of bifidobacteria and butyrate‐producing bacteria. This subsequently increases colonic butyrate levels (along with other SCFAs), which, as previously mentioned, enhance gut barrier function and influence metabolic hormones. Specific polyphenols, such as anthocyanins found in berries and chlorogenic acid present in coffee, influence the composition of microbiota and fermentation patterns, promoting microbial profiles associated with leanness. Fermented foods such as yogurt, kimchi, and kombucha offer live microbes and bioactive peptides that may reduce inflammation and promote the production of short‐chain fatty acids (SCFAs). For instance, the administration of oral butyrate in murine models not only diminished inflammation but also activated thermogenic gene expression in adipose tissue (Cook et al. 2021). In conclusion, fibers and components from fermented foods, while not directly involved in lipolysis, significantly influence the host's fat storage by enhancing short‐chain fatty acids and metabolite signals through the gut‐brain axis. These changes promote feelings of fullness, enhance insulin sensitivity, and increase energy expenditure.

### 3.5 Anti‐Inflammatory Compounds

A variety of bioactive compounds aimed at combating obesity operate by reducing the chronic inflammation associated with fat accumulation. Curcuminoids found in turmeric and gingerols present in ginger serve as quintessential illustrations. Curcumin derived from *Curcuma longa* demonstrates the ability to inhibit NFκB and inflammasome pathways in adipose tissue, leading to a reduction in TNFα, IL‐6, and various other cytokines (Moetlediwa et al. 2023). Clinical studies involving individuals with obesity indicate that curcumin supplementation significantly reduces serum levels of CRP, TNFα, and IL‐6 (Varì et al. 2021). In a similar manner, 6‐gingerol (ginger) decreases pro‐inflammatory adipokines (leptin, TNFα, MCP‐1) while increasing anti‐inflammatory adiponectin in adipose tissue (Hong, Um, et al. 2023). Marine omega‐3 fatty acids (EPA, DHA from oily fish) demonstrate anti‐adiposity effects by integrating into adipocyte membranes, which shifts the eicosanoid balance towards less inflammatory prostaglandins. This process reduces macrophage infiltration in fat and promotes fat oxidation through the increase of UCP1 and SIRT1 (Terzo et al. 2024). Berry flavonoids, such as resveratrol and anthocyanins, also reduce adipose inflammation through the inhibition of SIRT1/AMPK and NFκB pathways. Foods that are abundant in anti‐inflammatory phytonutrients, such as turmeric, ginger, berries, and fatty fish, play a crucial role in addressing obesity‐related immune activation. This, in turn, enhances insulin sensitivity and facilitates normal lipid metabolism.

These mechanism‐based classes collectively demonstrate the variety of food‐derived molecules (Figure 1)—from spices and herbs to fruits, vegetables, legumes, and marine products—that can influence fat metabolism. Through the elevation of energy expenditure, inhibition of fat‐cell formation, suppression of appetite, or mitigation of inflammation, each factor plays a role in influencing the balance between adiposity reduction and fat accumulation. In the realm of nutrition, the interplay of various bioactives (such as a spicy tea abundant in catechins combined with fiber and anti‐inflammatory herbs) could work together to foster a lean phenotype.

## 4 Mechanisms of Action of Bioactive Compounds in Fat Regulation

### 4.1 Cellular Regulators of Adipocyte Metabolism and Thermogenesis

At the cellular level, bioactive compounds modify energy balance through the activation of established metabolic sensors and transcriptional programs. The AMPK–SIRT1–PGC‐1α axis serves as a pivotal connection, facilitating the transition of cells from lipid storage to oxidation. For instance, numerous polyphenols and terpenoids stimulate AMPK (along with the NAD‐dependent deacetylase SIRT1), resulting in enhanced expression and activity of PGC‐1α. This promotes the formation of mitochondria and increases the expression of uncoupling protein‐1 (UCP1) in brown/beige adipocytes, consequently boosting thermogenic respiration and energy expenditure (Choi and Yu 2021). It has been observed that resveratrol and similar compounds enhance UCP1, PRDM16, and additional markers associated with brown fat in white fat depots, facilitating the process of browning via a SIRT1/PGC‐1α–dependent mechanism (Imamura et al. 2017). At the same time, the activation of AMPK enhances lipid breakdown: it phosphorylates acetyl‐CoA carboxylase and increases the expression of CPT1 (carnitine palmitoyltransferase‐1), facilitating fatty‐acid oxidation. A polyphenol‐rich extract, for instance, elevated AMPK and CPT1 levels while decreasing lipogenic transcripts in 3T3‐L1 adipocytes (Hong, Park, et al. 2023), indicating a switch to fat burning.

Bioactive compounds directly influence lipolysis. Through the activation of adrenergic or cAMP–PKA pathways, there is an enhancement of hormone‐sensitive lipase and perilipin phosphorylation, which facilitates the release of stored triglycerides. Certain flavonoids and alkaloids, such as caffeine, capsaicin, and menthol, stimulate thermogenic β3‐adrenergic pathways in adipocytes, thereby enhancing UCP1 and fuel oxidation. The enhancement of mitochondrial quality control is evident: the enhancement of PGC‐1α and SIRT1 leads to an increase in mitochondrial quantity and functionality within muscle and liver tissues, thereby facilitating overall fuel utilization and maintaining metabolic adaptability (Nikawa et al. 2021). Together, these cellular effects accelerate fatty acid oxidation and heat production (thermogenesis) and raise basal energy expenditure, countering adiposity.

### 4.2 Transcriptional Control of Adipocyte Differentiation and Lipid Storage

At the gene‐expression level, bioactives modify adipogenic transcription factors to decrease fat storage. PPARγ, a key regulator of adipocyte differentiation, along with its co‐activators C/EBPα and SREBP‐1c, plays a crucial role in the orchestration of lipogenesis and triglyceride accumulation. In cases of obesity, these factors are significantly elevated, promoting the increase of fat tissue. A variety of phytochemicals inhibit this adipogenic program. For example, EGCG (a catechin found in green tea) and flavonoids such as quercetin or hesperidin have demonstrated the ability to inhibit the expression of PPARγ and C/EBPα, along with SREBP‐1c and subsequent lipogenic enzymes (FAS, LPL) (Al‐Regaiey 2024). This transcriptional repression restricts the development of new adipocytes and the production of fatty acids. On the other hand, certain compounds can selectively influence PPAR isoforms: specific polyphenols enhance PPARα/δ to facilitate fatty acid breakdown, while simultaneously reducing PPARγ activity. For instance, kaempferol reduces the levels of PPARγ and SREBP‐1c while enhancing the expression of PPARα/δ target genes, which in turn promotes autophagy and facilitates fatty acid uptake (Kawser Hossain et al. 2016). Likewise, SIRT1 activation by resveratrol indirectly inhibits PPARγ‐driven adipogenesis (favoring lipolysis) (Al‐Regaiey 2024).

Furthermore, bioactives have the potential to increase the expression of genes related to lipolysis and oxidative processes. The outcome indicates a transition from lipid storage to mobilization: adipocytes exhibit heightened expression of β‐oxidation enzymes and mitochondrial proteins, alongside diminished expression of perilipin and adipokines that facilitate lipid accumulation (Hong, Um, et al. 2023). For example, adipose tissue treated with resveratrol shows reduced levels of C/EBPα and SREBP‐1c, while levels of PGC‐1α and CPT1α are elevated, indicating decreased lipogenesis and increased fat oxidation (Kazemi et al. 2025). In conclusion, the synergistic molecular effects of bioactives—stimulating AMPK/PGC‐1α, inhibiting PPARγ/C/EBP/SREBP, and enhancing mitochondrial functions—establish a cellular environment that promotes fat oxidation (thermogenesis and lipolysis) while preventing the formation of new fat cells (adipogenesis).

### 4.3 Gut–Brain Axis and Hormonal Modulation of Energy Balance

In addition to their direct cellular impacts, bioactive compounds interact with systemic endocrine and neural pathways to modulate adiposity. Compounds obtained from diet and their metabolites, such as polyphenols, fibers, and fatty acids, play a crucial role in modulating gut hormone secretion and neural communication with the brain. For instance, numerous polyphenols promote the secretion of glucagon‐like peptide‐1 (GLP‐1) from intestinal L‐cells and impede its breakdown, which in turn boosts satiety signaling and insulin secretion (Dominguez Avila et al. 2017). The effects of incretins are enhanced by the modulation of additional hormones: polyphenols and various phytochemicals frequently reduce ghrelin levels (the hunger hormone) while increasing adiponectin (the insulin‐sensitizing adipokine) (Balaji et al. 2016). High‐dose green tea polyphenols, for instance, inhibit ghrelin release and concomitantly raise adiponectin levels (Jin‐Feng et al. 2025), which helps blunt appetite and improve glucose uptake. A variety of compounds have the potential to enhance leptin signaling, thereby mitigating leptin resistance in obesity and restoring the balance of energy intake and expenditure feedback mechanisms.

The gut microbiota serves as an additional interface. The fermentation of fibers and polyphenols by bacteria produces short‐chain fatty acids (SCFAs; acetate, propionate, butyrate), which function as signaling molecules. Short‐chain fatty acids interact with G‐protein receptors (GPR41/GPR43) located on enteroendocrine and adipose cells, initiating hormone secretion and influencing metabolic processes. It is important to highlight that SCFAs enhance the secretion of GLP‐1 and peptide YY from the gut, which contributes to increased satiety and better glycemic control (van Son et al. 2021). These also engage vagal afferents, leading to a direct suppression of appetite. In adipose tissue, short‐chain fatty acid signaling via GPR43 has the potential to reduce fat accumulation and promote fat oxidation. For instance, propionate and butyrate stimulate intestinal gluconeogenesis and activate adipose AMPK, resulting in decreased lipid storage (Jiao et al. 2021). SCFAs also increase leptin production in adipocytes via GPR41, reinforcing satiety signals (May and den Hartigh 2021).

The neuroendocrine pathways work together to incorporate nutritional signals into the regulation of energy balance. By enhancing satiety hormones (GLP‐1, PYY, leptin) and diminishing orexigenic signals (ghrelin), bioactives contribute to a reduction in food intake. At the same time, enhanced insulin and adiponectin signaling optimizes nutrient distribution, directing a greater amount of glucose and lipids towards oxidation instead of storage. This gut–brain–hormone network complements cellular actions to promote adiposity reduction.

### 4.4 Context‐Dependent Actions: Obesity Versus Malnutrition/Cachexia

Significantly, the identical bioactive mechanism has the capacity to adjust to various nutritional conditions. In the context of obesity, characterized by caloric excess, inflammation, and insulin resistance, polyphenols and similar compounds play a role in suppressing lipogenesis and appetite, while also enhancing insulin sensitivity (Abiola et al. 2024). The reduction of tumor necrosis factor (TNF‐α) and interleukin‐6 (IL‐6) demonstrates anti‐inflammatory effects that help to restore the endocrine function of adipose tissue and promote the breakdown of fat. Conversely, in instances of undernutrition or cachexia, characterized by energy deficits and muscle wasting, analogous pathways may yield protective and anabolic effects. For instance, the activation of the AMPK–SIRT1 axis and its subsequent mitochondrial programs enhances muscle oxidative capacity and viability. Resveratrol, known for its ability to inhibit NF‐κB and the ubiquitin–proteasome pathways, plays a crucial role in preserving muscle mass by blocking catabolic signaling (Nikawa et al. 2021). This mechanism operates by activating the Akt/mTOR pathway to enhance protein synthesis while simultaneously inhibiting inflammatory proteolysis in myocytes (Kaur et al. 2024). Thus, in cachexia, the net effect is tissue protection rather than breakdown.

Similarly, the actions that enhance insulin sensitivity offer twofold advantages. In cases of obesity, they mitigate hyperinsulinemia and avert excessive adipose accumulation; in instances of malnutrition, they enhance nutrient absorption and prevent muscle glucose deficiency. For example, compounds that elevate adiponectin enhance peripheral glucose utilization in both scenarios. However, they reduce fat storage in obesity while simultaneously preserving glucose for vital functions during starvation.

In conclusion, bioactive compounds influence key metabolic pathways (AMPK, PPARγ, UCP1, SREBP‐1, C/EBPα, PGC‐1α) to shift the equilibrium between fat accumulation and reduction (Hong, Park, et al. 2023). By means of synchronized cellular and systemic mechanisms—including increased thermogenesis and lipolysis, as well as gut–brain hormone signaling—they promote energy expenditure and nutrient distribution. Importantly, their effects exhibit flexibility: mechanisms that reduce inflammation and enhance insulin sensitivity can facilitate weight loss in a calorie‐dense environment while also maintaining lean mass and energy reserves during periods of reduced intake. The mechanistic insights provide a foundation for the therapeutic potential of combinatorial bioactive interventions aimed at rectifying energy imbalances across various metabolic conditions.

## 5 Synergistic Nutrient Pairings

**FIGURE 2:**
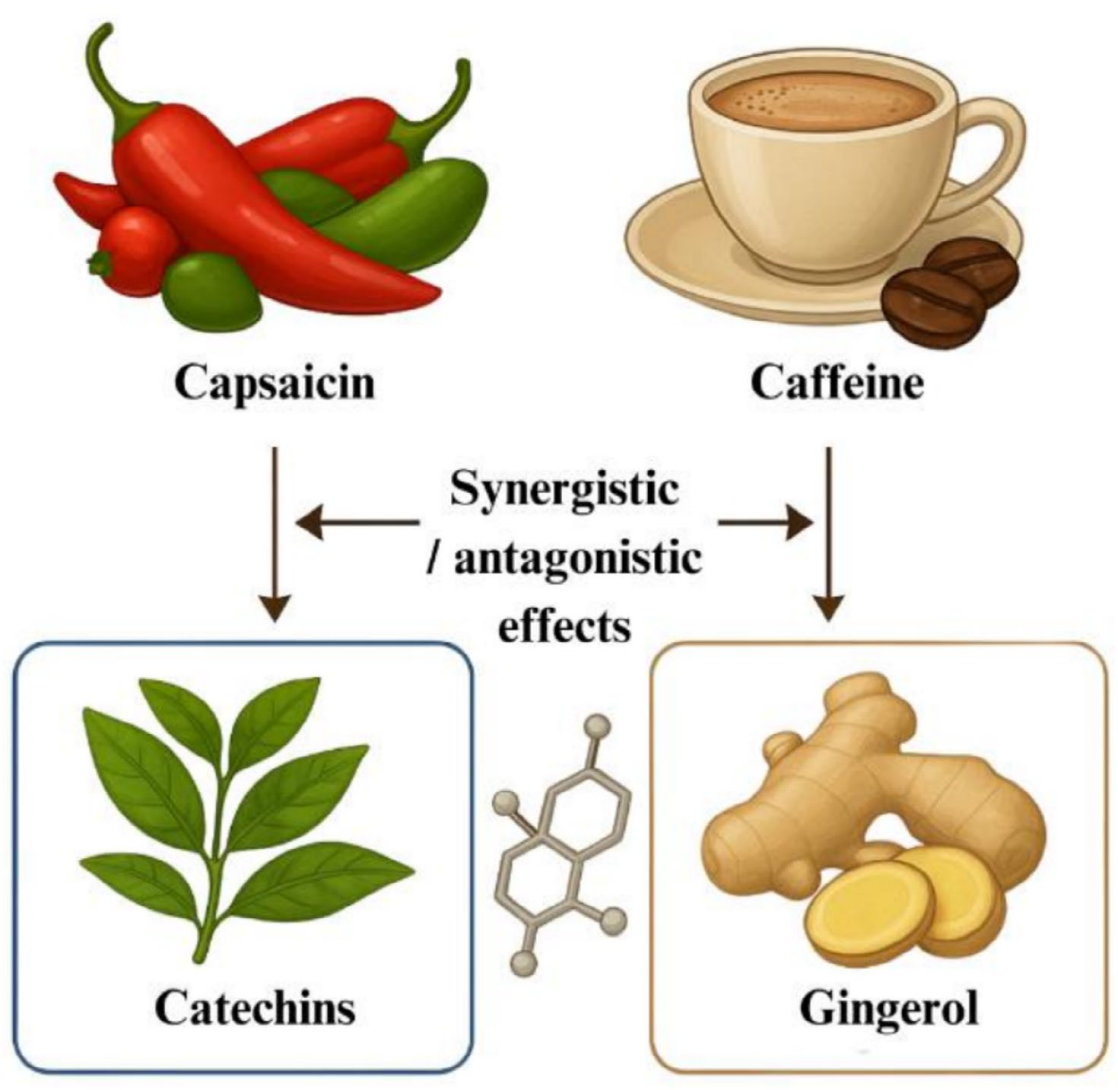
Synergistic pairing of natural‐occurring compounds.

Fat modulation frequently results from the synergistic interactions of multiple bioactive compounds rather than from individual substances alone. In the natural world, fruits and botanicals are often found together, offering a combination of fiber and polyphenols, such as in berries and pomegranates. Similarly, beverages like green tea merge catechins, specifically EGCG, with caffeine and L‐theanine (Figure 2).

The natural combinations and carefully crafted blends, such as berberine combined with resveratrol and polyphenol‐probiotic synbiotics, have the potential to enhance the impact on adiposity beyond what each individual component can achieve on its own. For example, the combination of mulberry‐leaf fiber and its polyphenols resulted in more significant weight loss and alterations in gut microbiota compared to each component individually (Nemzer et al. 2025). In a similar vein, low‐dose EGCG combined with caffeine demonstrated “synergistic anti‐obesity effects comparable to those of high‐dose EGCG,” influencing gut flora (↑Bifidobacteria, SCFA) and bile‐acid signaling to enhance fat oxidation (Zhu et al. 2021). Notably, certain synergies may facilitate weight gain: the integration of nutrient‐dense foods or supplements, such as high‐protein sources combined with leucine and vitamin D, promotes the increase of lean mass in individuals experiencing sarcopenia or cachexia (Chang and Choo 2023).

A variety of mechanisms contribute to this synergy. Enhancement of bioavailability: Specific adjuvants significantly improve absorption. Notable instances involve piperine in conjunction with curcumin—administering 2 g of curcumin alongside 20 mg of piperine resulted in an approximate 20‐fold increase in the blood AUC of curcumin compared to curcumin alone (Tabanelli et al. 2021)—and EGCG with L‐theanine or lipid complexes, which form soluble micelles, boosting EGCG's uptake (Liu et al. 2024).

Multi‐pathway targeting: various bioactives can simultaneously engage different metabolic pathways. Combining berberine and resveratrol resulted in a more significant reduction in blood lipids and adipocyte fat compared to using either compound individually (Zhu et al. 2018). Resveratrol, known for its role as a SIRT1 activator, increased intracellular levels of berberine and boosted LDL‐receptor expression, thereby amplifying the effects of berberine. Microbiota modulation: Fiber and polyphenols frequently function as synbiotics. Their simultaneous presence can alter gut ecology and fermentation processes. For instance, the combination of prebiotic fibers and certain Lactobacilli strains effectively diminished weight gain in mice by enhancing fecal SCFAs and modifying bile acids (Apalowo et al. 2024). Likewise, diets rich in bound polyphenols (often attached to fiber) promote beneficial bacteria and SCFA production (Nemzer et al. 2025).

These synergies are relevant in the contexts of both adiposity reduction and fat accumulation. Numerous combinations enhance fat loss by boosting thermogenesis, lipolysis, or satiety: For instance, the combination of EGCG from green tea and caffeine, when consumed in the morning or prior to exercise, optimizes fat oxidation (Roberts et al. 2021; Rothenberg et al. 2018); berry fiber + polyphenols (with meals) blunt glucose spikes and promote fullness via gut fermentation. On the other hand, support for weight gain frequently involves the simultaneous provision of protein or energy along with anti‐catabolic agents: Formulas that are high in protein and enriched with leucine and vitamin D, to be taken with meals or at bedtime, maintain muscle mass and body mass index in cachexia (Son et al. 2020). Timing is crucial: fat‐oxidative combinations (such as caffeine and EGCG) tend to be most effective when taken pre‐workout or in the morning, while fat‐gain enhancers (like protein and omega‐3) are better suited for consumption with meals or at bedtime to synchronize with anabolic cycles.

In conclusion, leveraging nutrient synergy through enhanced delivery, multi‐target action, or microbiome interactions can amplify effects on body fat beyond what isolated compounds can achieve. The following table (Table 1) illustrates representative synergistic pairings, their primary direction of effect, and optimal timing of intake:

**TABLE 1:** Synergistic combinations for fat modulation: optimal timing and functional outcomes.

Bioactive combination	Direction	Timing (optimal)	References
EGCG + caffeine (green tea extract)	Adiposity reduction	Morning/pre‐exercise	(Liu et al. 2024; Zhu et al. 2021)
Fiber + polyphenols (berry‐rich foods)	Adiposity reduction	With meals	(Nemzer et al. 2025)
EGCG + L‐theanine (green tea)	Adiposity reduction	Morning/with tea	(Liu et al. 2022)
Curcumin + piperine	Adiposity reduction	With meals	(Hosseini et al. 2023; Shoba et al. 1998)
Resveratrol + berberine	Adiposity reduction	With meals	(Zhu et al. 2018)
Probiotic + prebiotic (oat β‐glucan)	Adiposity reduction	With meals	(Apalowo et al. 2024)
Capsaicin + eggs (protein)	Adiposity reduction	Breakfast	(Smeets et al. 2013)
Whey protein + leucine + vitamin D	Fat accumulation	Post‐exercise/bedtime	(Chang and Choo 2023)
Omega‐3 PUFA + protein (±vitamin D)	Fat accumulation	With meals	(Son et al. 2020)

## 6 Clinical Evidence in Humans

Clinical trials and meta‐analyses are progressively demonstrating modest effects of food‐derived bioactives on fat mass across various adult populations. In individuals with overweight or obesity, supplements rich in polyphenols (such as green tea catechins, curcumin, resveratrol, and berry extracts) have demonstrated statistically significant, albeit modest, reductions in body weight, BMI, and waist circumference (Asbaghi et al. 2024; Unhapipatpong et al. 2023). An umbrella meta‐analysis encompassing 50 randomized controlled trials revealed that daily doses of curcumin (turmeric) resulted in a modest reduction in body weight (mean difference = approximately −0.6 kg) and BMI (−0.24 kg/m^2^) over several weeks, with formulations that enhance bioavailability producing slightly greater reductions (Unhapipatpong et al. 2023). Similarly, green tea extract (catechins = approximately 500–1000 mg/day) reduced weight (~0.6 kg) and body fat (%) relative to placebo (Samavat et al. 2016). A thorough meta‐analysis of green tea trials indicated that supplemental catechins notably reduced body mass (WMD = approximately –0.64 kg), BMI, and fat percentage, particularly in short‐term (≤ 12 weeks) studies involving younger adults (Asbaghi et al. 2024). Polyphenol interventions typically demonstrate a range of metabolic effects, including antioxidant activity, AMPK activation, modulation of adipokines, and interactions with microbiota; however, the outcomes can differ significantly. Participants with greater baseline adiposity or younger age often experience more significant benefits (Zhang, Balasooriya, et al. 2023). For instance, subgroup analyses indicate greater adiposity reduction with polyphenols in obese subjects and in trials of longer duration (≥ 3 months).

Alkaloids and associated phytochemical stimulants exhibit indications of weight loss. Caffeine, whether sourced from coffee, tea, or supplements, has been shown to modestly enhance thermogenesis and fat oxidation. Meta‐analyses of randomized controlled trials indicate that increased caffeine consumption is linked to progressive reductions in weight and fat: each doubling of the dose corresponds to approximately a 20% greater decrease in weight, BMI, or fat mass (Tabrizi et al. 2019). In practical terms, randomized controlled trials indicate that regimens containing caffeine result in modest reductions in body weight and body fat when compared to decaffeinated controls, with average decreases of approximately 1%–2% in total weight. In a similar vein, capsaicin and capsinoids, the spicy elements found in chili peppers, exhibit small yet noteworthy effects. A recent meta‐analysis encompassing 15 randomized controlled trials revealed that capsaicin consumption led to a reduction in weight by approximately 0.5 kg, a decrease in BMI by around 0.25 kg/m^2^, and a reduction in waist circumference by about 1.1 cm among overweight adults (Zhang, Zhang, et al. 2023). The safety of these thermogenic compounds at dietary doses seems to be established, yet their effectiveness is relatively modest and frequently varies with dosage. It is important to recognize that outcomes may be influenced by variations in trial designs, including differences in populations, endpoints, and comparator diets (Hillsley et al. 2022). A review of resveratrol trials in obesity revealed significant inconsistencies in dosage, formulation, and endpoints, complicating the ability to compare findings across studies. In conclusion, stimulant phytochemicals such as caffeine and capsaicin result in modest weight and fat loss among individuals with high adiposity, although the effects can differ based on dosage and accompanying diet and exercise regimens.

Dietary fiber and prebiotics have demonstrated significant effectiveness in promoting fat loss among individuals with excess weight. Meta‐analyses of isolated fibers indicate reliable reductions in both weight and waist circumference. Psyllium (10–15 g/day) led to a reduction in body weight of approximately 2.1 kg and a decrease in BMI of around 0.8 kg/m^2^ over a period of about 5 months in individuals classified as obese (Gibb et al. 2023). Inulin‐type fructans (inulin/oligofructose 10–21 g/day) noted reductions in weight (~1 kg), BMI (−0.4 kg/m^2^), fat mass, and waist circumference observed in a combined trial (Reimer et al. 2024). The advantages of fiber stem from increased feelings of fullness, slower absorption rates, and the influence on gut microbiota composition. A recent trial involving obese individuals, specifically chosen for FTO/MC4R genetic variants, demonstrated that a 6‐month intervention with a fiber blend (glucomannan, inulin, psyllium) resulted in approximately 5% weight loss and around 13% reduction in fat mass compared to a placebo (Pokushalov et al. 2024). Notably, the response differed according to genotype: individuals with obesity‐risk alleles experienced more significant losses. Consequently, the impact of fiber on weight management is typically dependable; however, its effectiveness varies based on dosage (usually ≥ 10 g prior to meals) and personal characteristics (such as gut flora and genetic factors).

Bioactive peptides and proteins play a crucial role in preventing adiposity reduction, specifically in preserving or gaining lean mass among malnourished or sarcopenic patients. In populations that are elderly or experiencing wasting, the inclusion of high‐quality protein with targeted amino acid profiles has the potential to enhance lean mass and may also aid in overall weight gain. For instance, the integration of resistance training with collagen or whey peptides significantly enhanced body composition in sarcopenic adults: one study reported a +4.2 kg increase in fat‐free mass (compared to +2.9 kg with placebo) and notable strength gains over a 12‐week period (Zdzieblik et al. 2015). The use of leucine metabolites (β‐hydroxy‐β‐methylbutyrate), whey protein hydrolysates, or essential amino acids frequently enhances muscle protein synthesis in frail or sarcopenic older adults. A systematic review indicated that polyphenol supplements, such as isoflavones and curcumin, notably enhanced muscle mass in older patients with sarcopenia, with a standardized mean difference of approximately 1.5 (Medoro et al. 2024), though effects on strength were less clear. In wasting syndromes such as cancer cachexia, clinical trials involving high‐protein, calorie‐dense nutritional formulas—often enhanced with ω‐3 fatty acids, arginine, HMB, and glutamine—have occasionally resulted in modest weight gain and retention of lean mass (Braha et al. 2022). Pooled trials involving cancer patients indicate that supplements such as EPA (fish oil), arginine, glutamine, or HMB have the potential to enhance body weight and mitigate muscle loss. In summary, specific interventions involving peptides or proteins have the potential to mitigate catabolic weight and fat loss, particularly when paired with exercise; however, further research is required to gain a clearer mechanistic understanding.

The outcomes across various intervention types exhibit significant variability. Responses are influenced by various factors, including dosage and formulation, with enhanced‐bioavailability curcumin demonstrating superior performance (Unhapipatpong et al. 2023), baseline adiposity, age, sex, and microbiome composition. Many meta‐analyses find stronger effects in younger or obese subgroups (Zhang, Balasooriya, et al. 2023). For instance, the advantages of green tea catechins were primarily observed in short‐term studies (≤ 12 weeks) with participants under 50 years of age (Asbaghi et al. 2024). A meta‐analysis on polyphenols indicated that weight loss was substantial among Asian and participants under 50 years, while it was minimal in older Western groups (Nikpayam et al. 2025). The gut microbiota can significantly influence efficacy: prebiotic fibers alter microbial populations (such as enhancing Bifidobacteria), which may subsequently affect fat metabolism and inflammation. Research indicates that inulin‐type fibers can decrease fat mass while potentially maintaining lean mass through metabolites derived from microbiota. Ultimately, there is significant variability in trials—different studies employ diverse outcomes (weight versus fat mass), comparators, and co‐interventions—which means that some reported “null” results might be indicative of study design issues rather than an absence of biological effect.

In conclusion, recent robust evidence indicates that bioactives derived from food can promote modest fat loss in individuals with excess weight and assist in the gain of fat and lean mass in those who are undernourished. Polyphenols found in green tea, turmeric, berries, and coffee, along with caffeine and chili compounds, contribute to modest yet notable reductions in fat associated with obesity. In contrast, fibers and prebiotics offer more substantial weight loss and metabolic advantages. Conversely, interventions targeting wasting conditions, such as high‐protein or amino acid formulas, ω‐3 fatty acids, and polyphenols, demonstrate promise in mitigating weight loss or enhancing lean mass. Future trials should consider individual differences such as dose, age, adiposity, and gut microbes, while employing standardized outcomes to determine which bioactives or combinations provide significant clinical benefits in human adiposity and cachexia.

## 7 Translational and Personalized Nutrition Applications

### 7.1 Population‐Level Interventions

At the population level, efforts in translation emphasize the integration of bioactive‐rich foods into standard dietary practices and policies. For instance, there is ongoing development of functional foods and fortified products, such as fiber‐ or polyphenol‐enriched beverages and probiotic yogurts, aimed at providing metabolic benefits on a large scale. Comprehensive dietary recommendations are progressively highlighting the importance of plant‐based, nutrient‐rich diets; however, actual compliance and effectiveness in practice continue to be constrained. In a recent analysis, it was observed that conventional “one‐size‐fits‐all” recommendations exhibit significantly low compliance and overlook considerable inter‐individual variability (Bermingham et al. 2024). Indeed, even a highly motivated population randomized trial found that standard USDA guideline advice produced only modest cardiometabolic improvements, whereas a tailored diet (utilizing individual post‐meal glucose, lipid, and microbiome profiles) yielded significantly better outcomes. The observations highlight the complexities involved in population strategies: while it is possible to promote bioactive‐rich foods on a large scale, generic methods will only produce minimal benefits unless they consider the diversity among users.

### 7.2 Personalized Strategies and Digital Technologies

At the individual level, nutrition can be tailored using personal data and advanced technology. Key avenues include:

### 7.3 Genetic Profiling (Nutrigenomics)

People possess genetic variations that influence how nutrients are metabolized and how fat is stored. Organizations currently provide SNP‐based dietary profiles (such as “fat‐responder” versus “carb‐responder” genotypes) to assist in determining macronutrient distribution. A recent high‐profile trial involved genotyping overweight adults for 10 obesity‐related loci. Participants were assigned to either a high‐fat or high‐carb diet, which was categorized as either concordant or discordant with their genetic profiles (Höchsmann et al. 2023). Following a 12‐week period, there was no notable difference in weight loss observed between the genotype‐matched and mismatched groups. This indicates that although genetics play a role in metabolism, relying solely on single‐gene panels or basic genetic models is inadequate; truly effective precision diets probably necessitate the incorporation of multiple genes along with additional elements.

### 7.4 Gut Microbiome Profiling

The gut microbiota significantly influences the energy balance and bioactive metabolism of the host. Emerging companies such as DayTwo, Viome, and ZOE are now analyzing fecal microbiomes to forecast personalized glycemic or lipid reactions to various foods. These services frequently employ machine‐learning models to convert a microbiome snapshot into tailored food and supplement suggestions. Nonetheless, specialists warn that this field is still in its early stages (Simon et al. 2023). A recent review highlighted the absence of standardization in microbiome tests, the ambiguity surrounding what constitutes a “healthy” reference, and the significant variability in interpersonal microbiota. In practice, altering a single variable (such as a particular food) typically results in only a slight effect on the overall gut ecosystem and health. Therefore, advice informed by the microbiome demonstrates potential, yet it requires validation through larger trials. A study published in Nature involving pregnant women indicated that incorporating microbiome data into a postprandial glucose model resulted in only a marginal improvement in prediction (*R* ^2^ increased from approximately 0.50 to about 0.52) (Popova et al. 2025). Ongoing investigations are shedding light on the microbial signatures that reliably forecast responses to specific bioactives; however, it is important to exercise caution before drawing conclusions from individual test outcomes.

### 7.5 Wearable Biomarker Tracking

Contemporary health wearables—particularly continuous glucose monitors (CGMs) and activity trackers—provide immediate insights into the impact of diet and exercise on physiological responses. Innovative initiatives are combining continuous glucose monitoring data with mobile applications to develop adaptive, evidence‐based dietary plans. An open‐label trial involving 2200 adults was conducted, where participants utilized continuous glucose monitors (CGMs) for a duration of 28 days. During this period, they recorded their meals and physical activity. A connected application was employed to analyze glucose patterns in relation to macronutrient intake, glycemic index/load, and levels of exercise. This intervention demonstrated notable enhancements in glucose regulation, weight management, and dietary quality when compared to baseline measurements (Zahedani et al. 2023). Significantly, there was a reduction in average blood glucose excursions, a decrease in average weight (particularly among overweight individuals), and a transition towards meals that are higher in fiber and protein. This indicates that integrating bioactive‐guided recommendations with ongoing biomarker monitoring and tailored feedback may encourage healthier decision‐making and improved fat management. Recent investigations (e.g., employing CGM in non‐diabetics) are now validating that real‐time metabolic monitoring can function as an “early warning” and behavioral signal for individuals.

### 7.6 AI‐Driven Meal Planning

Artificial intelligence is utilized to create customized meal plans that align with specific bioactive objectives. Innovative recommendation systems leverage advanced deep neural networks that are trained on comprehensive nutritional databases and dietary guidelines to curate personalized weekly menus for individual users. A recent study published in Scientific Reports detailed a diet application that integrates expert guidelines from EFSA and WHO with a deep generative model. This AI‐driven tool generates weekly meal plans tailored to caloric requirements and nutrient balance, utilizing a large language model, such as ChatGPT, to enhance the variety of recipe choices (Papastratis et al. 2024). This method facilitates the swift creation of tailored menus that are not only enjoyable (based on actual recipes) but also in harmony with the user's health objectives. Initial evaluations indicated that these AI systems are capable of achieving or surpassing the performance of conventional rule‐based planners in terms of precision and user contentment. With advancements in AI chatbots, there is potential for them to offer on‐demand nutrition coaching, addressing inquiries related to recipes or suggesting ingredient substitutions that are abundant in essential bioactives. Nonetheless, meticulous curation is essential: unregulated outputs from language models can occasionally propose nutritionally questionable alternatives; thus, the most effective systems integrate AI innovation with established dietary guidelines (EFSA Panel on Dietetic Products 2012; World Health Organization 2021).

### 7.7 App‐Based Nudging (JITAI)

Ultimately, mobile applications provide timely behavioral prompts that promote healthier decision‐making. These could employ push notifications or text messages strategically timed for moments of need, such as pre‐meal reminders to select a polyphenol snack or celebratory feedback following a healthy meal. The notion of JITAIs (Just‐In‐Time Adaptive Interventions) is increasingly being recognized in weight‐loss trials (Nezami et al. 2025). For instance, the AGILE trial is rigorously examining various combinations of coaching messages, self‐monitoring features, and adaptive goal‐setting through applications. In application, an application could analyze a grocery barcode or meal image and promptly recommend an adjustment (“Incorporate spinach for enhanced fiber”) or promote the addition of an anti‐inflammatory herb into a dish. While currently in the phase of clinical investigation, these digital nudges have the potential to support dietary changes in everyday settings, where self‐control frequently falters.

In practice, numerous elements are currently aligning. Innovative personalized nutrition platforms integrate multi‐omic data alongside AI‐driven coaching methodologies. An application could analyze your genetic risk score (such as ApoE status and FTO genotype), gut microbiome profile, recent glucose logs, and even your sleep and activity patterns. It would then algorithmically process all this data to evaluate and score foods and meals tailored to you. Individuals are provided with a daily “food index” or tailored menu that evolves in response to incoming data. A number of commercial services currently adopt this approach, such as ZOE and DayTwo, which utilize microbiome and blood markers, while others like Nutrigenomix rely on genetic panels. In a similar vein, certain companies promote “metabolic cocktails”—capsules or shakes infused with recognized bioactives (such as green tea catechins, curcumin, berberine, etc.)—aimed at specific subgroups (for instance, “gut health” blends or “fat‐burning” formulas). Although the popularity of direct‐to‐consumer options is significant, their actual clinical value remains under investigation. Ongoing controlled trials of polyphenol supplements and synbiotic beverages are focused on translating mechanistic research into validated therapies.

In conclusion, the translational pathway is evolving to be more integrative: data on bioactives is being incorporated into digital tools and behavior change initiatives. Population‐level measures, such as functional‐food policy and updated guidelines, establish a wide‐ranging context; however, digital health platforms are progressively customizing that advice to align with individual biology and lifestyle. In the future, we expect that diets guided by AI, utilizing insights from genetic and microbial testing, will enhance conventional public health recommendations. Ongoing advancements—like integrating the timing of meals with circadian rhythms, continuous biomarker monitoring, and enhanced omics—will enhance the personalization of interventions. It is crucial that the distinct “metabolic phenotype” of each individual, encompassing factors such as gut microbiota, genotype, chronotype, and activity patterns, is incorporated into forthcoming nutrition guidelines. This method holds the potential to enhance the effectiveness of bioactive compounds for adiposity reduction and general well‐being in each person, moving beyond mere averages.

## 8 Conclusion

Dietary bioactive compounds serve as a significant, yet often overlooked, avenue for influencing the balance of adipose tissue in both adiposity reduction and fat accumulation scenarios. The compounds influence various cellular regulators such as AMPK and PPARγ, as well as systemic pathways that include the gut–brain axis and microbiota. Their pleiotropic effects can be utilized for therapeutic and preventive measures. Importantly, the synergistic combinations—whether found naturally in foods or designed in functional products—often surpass the effectiveness of single‐agent approaches. Clinical trials have demonstrated consistent yet modest effects on fat mass across both obese and undernourished groups, with outcomes influenced by factors such as age, adiposity, genetics, and microbiome composition. New digital tools, including AI‐based meal planning and biomarker‐informed personalization, provide opportunities to convert mechanistic insights into tailored dietary strategies. As the field progresses, it will be crucial to tackle the challenges of bioavailability, standardization of trials, and ensuring equitable access. Ultimately, the integration of food science, systems biology, and precision technology establishes bioactives as crucial influencers of human metabolic resilience. Future research should focus on: (i) enhancing the bioavailability of bioactives, (ii) creating synergistic blends based upon mechanistic understanding, (iii) implementing long‐term standardized randomized controlled trials to evaluate efficacy and safety, and (iv) incorporating AI‐driven personalization to customize interventions for diverse populations.

## Author Contributions

**Mohamed Ibrahim Younis:** writing – original draft, formal analysis, data curation, visualization, methodology, conceptualization, software, writing – review and editing, resources. **Rawaa H. Tlay:** methodology, conceptualization, writing – review and editing, writing – original draft, formal analysis, data curation, visualization, software, resources, supervision. **Ammar B. Altemimi:** formal analysis, data curation, visualization, methodology, conceptualization, writing – review and editing, supervision. **Zheng Ruan:** methodology, conceptualization, writing – review and editing, supervision. **Tarek Gamal Abedelmaksoud:** writing – original draft, formal analysis, data curation, visualization, methodology, conceptualization, software, writing – review and editing, supervision, resources.

## Conflicts of Interest

The authors declare no conflicts of interest.

## Data Availability

Data will be made available on request.
